# Pharmacokinetics and toxicity of subcutaneous administration of carboplatin in poloxamer 407 in a rodent model pilot study

**DOI:** 10.1371/journal.pone.0186018

**Published:** 2017-10-05

**Authors:** Marije Risselada, Keith E. Linder, Emily Griffith, Brittney V. Roberts, Gigi Davidson, William C. Zamboni, Kristen M. Messenger

**Affiliations:** 1 Department of Clinical Sciences, College of Veterinary Medicine, North Carolina State University, Raleigh, North Carolina, United States of America; 2 Department of Veterinary Clinical Sciences, College of Veterinary Medicine, Purdue University, West Lafayette, Indiana, United States of America; 3 Department of Population Health and Pathobiology, College of Veterinary Medicine, North Carolina State University, Raleigh, North Carolina, United States of America; 4 Department of Statistics, College of Agriculture and Life Sciences, North Carolina State University, Raleigh, North Carolina, United States of America; 5 UNC Eshelman School of Pharmacy, UNC Lineberger Comprehensive Cancer Center, University of North Carolina at Chapel Hill, Chapel Hill, North Carolina, United States of America; 6 CPS, College of Veterinary Medicine, North Carolina State University, Raleigh, North Carolina, United States of America; 7 Department of Molecular Biomedical Sciences, College of Veterinary Medicine, North Carolina State University, Raleigh, North Carolina, United States of America; Shiraz University, ISLAMIC REPUBLIC OF IRAN

## Abstract

The objectives of this study were to assess the pharmacokinetics and safety of subcutaneously delivered carboplatin in poloxamer 407 in rats. Carboplatin (5mg/rat) in 0.5ml poloxamer 407 (1.0 ml total volume) was administered subcutaneously in a right subcutaneous perineal incision in all 12 treatment rats. Three control rats received 1.0 ml of poloxamer 407. Total platinum was measured in plasma q24hrs from 0 to 168hrs. Protein-unbound platinum was measured in plasma at 168hrs. After sacrifice on day 7, total platinum was determined in wound bed muscle. Platinum concentrations in all samples were measured by ICP-MS. Wounds were visually assessed daily for 7 days. Perineal tissues (full wound bed including muscle, subcutis, skin) were assessed histologically and scored. Total platinum was detectable in plasma from 24 to 168 hrs. Total plasma platinum AUC and C_max_ were 9,165.3 ng/mL•h and 129.4 ng/mL. Day 7 total platinum concentration in muscle was approximately 10-fold higher than total plasma platinum concentration. No unbound platinum was detected in plasma samples at 168 hours. No wound healing complications were detected at any time point, nor was tissue necrosis observed histologically. The results of this study suggest that subcutaneous carboplatin in poloxamer 407 can be used in vivo providing direct tissue exposure to carboplatin without significant local effects or systemic absorption and without wound healing complications.

## Introduction

There is a need for adjunctive means of local tumor control following surgery in anatomically challenging locations. One such tumor is an apocrine gland adenocarcinoma of the anal sac (AGASACA). These are commonly occurring, locally invasive tumors of the anal gland/anal sac complex. Due to the need to preserve local vital structures, residual tumor cells frequently remain following excision, leading to recurrence in 29% to 45% of patients [1–3]. Radiation therapy is currently recommended for postoperative treatment of residual microscopic disease, but is problematic due to the high cost and incidence of adverse effects, in particular locally occurring side effects [4]. The role of systemic chemotherapy in local disease management is not well established, and such therapy is similarly associated with substantial acute adverse effects and cost. A chemotherapeutic agent that can be instilled into the wound bed at the time of surgery could considerably improve local disease control.

Poloxamer 407 is a nonionic polyoxyethylene-polyoxypropylene-polyoxyethylene (PEO-PPO-PEO) compound, which is unique in that it undergoes reverse gelatination, i.e. it changes from a liquid to a gel when it warms to temperatures higher than 25°C (including body temperature) [5,6]. This property has been shown to make poloxamer 407 a good carrier medium for prolonged local antifungal therapy [5]. It has also shown to have sustained delivery *in vivo* of hydrophilic macromolecules after intramuscular injections in rats [7]. It might show similar properties for subcutaneously administered chemotherapeutics. Prior studies have shown that poloxamer 407 by itself does not have adverse tissue effects in rats [7–9], and that poloxamer 407 will release 99% of the carboplatin as opposed to other carriers [10].

Carboplatin is a platinum chemotherapeutic agent that is used to systemically treat a variety of tumors, such as osteosarcoma and ASAGACA [2,3,11–16], and has been shown to be stable in poloxamer carriers [17,18] and fully release carboplatin [18] however information is not available about the stability of the combination of carboplatin and poloxamer gel *in vivo* or whether extensive local tissue damage occurs secondary to subcutaneous administration of this drug-poloxamer combination.

The objectives of this study were to 1) obtain pharmacokinetic data after perineal subcutaneous delivery of carboplatin in poloxamer copolymer gel, and 2) assess tissue safety and tissue effects of subcutaneously delivered carboplatin in poloxamer copolymer gel.

Our hypotheses were that in rats 1) carboplatin in poloxamer copolymer gel (carbo-poloxamer) instilled into perineal subcutaneous tissues will provide a sustained local presence of carboplatin leading to high local tissue concentrations but low plasma concentrations, and 2) carbo-poloxamer at a concentration of 5mg/ml does not cause local tissue necrosis.

## Materials and methods

The carboplatin-poloxamer mix was compounded by mixing an equal volume of 10 mg/ml carboplatin^a^ (Carboplatin 10mg/mL, Hospiram Inc., Lake Forest, IL Carboplatina 10mg/ml (5 mg) with poloxamer copolymer gel^b^ (0.5 mL) (Pluronic F127, poloxamer 407 (25% w/v;) 25% gel, Professional Compounding Centers of America, Houston, TX) under sterile conditions approximately 15 minutes prior to use.

### Animals

The study was approved by the Institutional Animal Care and Use Committee at North Carolina State University (#14-080-B). Fifteen purpose bred female Sprague Dawley rats (Charles River, Wilmington, MA, US) were used in the study: three control rats without preplaced jugular vein catheters and two cohorts (n = 6) of treated study rats with preplaced jugular vein catheters. Animals were housed in a dedicated room with a 12:12 light: dark cycle in individual see through cages with a stainless steel lid with embedded filter with free access to water and food. Bedding consisted of Bed-o’Cobs ¼” (The Andersons, Inc Maumee, OH) and was changed on a weekly basis or earlier if needed. Environmental enrichment was provided and consisted of Nylabones and autoclaved cardboard tubes. Catheter maintenance was performed per vendor’s (Charles River, Wilmington, MA) recommendations and guidelines. This catheter was secured in place by skin clips and plugged with a blunt ended metal tip. Briefly: The distal end of the catheter could be retrieved after prepping the site and loosening or removing the clips. A heparin locking solution was used to prevent clotting and was removed prior to obtaining the sample. A fresh amount of locking solution was instilled after obtaining the sample and administering a volume of saline similar to the blood sample, after which the catheter was reinserted subcutaneously and secured in place. In all rats, a right perineal subcutaneous incision was made under general anesthesia (Isoflurane (Piranal Healthcare, Piranal Enterprises Ltd, Andhra Pradesh, India) in 100% O2 for box induction and mask maintenance), with a large enough subcutaneous pocket to hold 1ml of the compound, after which the incision was sutured routinely. Analgesia was provided by administration of meloxicam Q 24hrs (1mg/kg PO or SC)(Metacam, Boehringer Ingelheim, St Joseph, MO) the day prior, the day of and the day following surgery, and an additional injection of buprenorphine (Endo Pharmaceuticals, Spring Valley, NY) (0.03mg/kg SC) was provided prior to recovery.

### Pharmacokinetics and local tissue toxicity

All rats in the two treatment cohorts received 1 ml of 5 mg/ml carboplatin-poloxamer mix instilled directly into the surgically created subcutaneous wound bed. The carboplatin-poloxamer mix was instilled in the subcutaneous pocket and the wound was sutured routinely in two layers after allowing the compound to gel *in situ*. In the treatment group, rats were divided in 2 cohorts: one cohort (n = 6) only received carboplatin-poloxamer (CP treatment cohort), the second cohort (n = 6) received carboplatin-poloxamer and had a microdialysis (μD) probe (LM-5 Linear microdialysis probe, BASi Inc., West Lafayette, IN) implanted for an unrelated study (CP-μD treatment cohort). This microdialysis probe was placed in the wound bed, secured with a suture in the wound and tunneled subcutaneously to exit dorsally in the cervical area. At 24 hours after surgery all probes were removed or cut flush at the cervical exit area if resistance was felt during removal. No dialysates were included or reported in this study. The control rats (n = 3) all received 1 ml of poloxamer copolymer gel only in the wound. In addition, two of the three control rats received a μD probe in the wound bed as described above, while the third control rat did not receive a μD probe. No control rat had a microdialysis sample collected at any time point.

### Sample collection

The pre-placed jugular vein catheters were used to obtain daily blood samples (q24hrs) for total plasma Pt concentration (0.3 ml) at the time of wound assessment in all rats of all three treatment cohorts. On day 7, blood (3 ml) (all control and treatment rats) for the 168 hours total plasma Pt and unbound plasma Pt concentration as well as tissue samples (wound bed for total tissue platinum concentration [CP cohort]; wound bed, colon and contralateral perineal area for histology [all rats]) were taken after sacrificing the rat. No microdialysate samples were included in the study.

### Analytical Studies

All plasma and muscle samples were stored at -80°C until analyzed for platinum (Pt) using a validated inductively-coupled plasma mass spectroscopy (ICP-MS) method as previously reported [19]. Plasma samples were processed to measure total Pt at all time points and additionally protein-unbound Pt at 168 hours. Muscle samples were processed to measure total Pt as previously described [20]. Briefly, muscle tissue and plasma samples were digested in concentrated (70%) HNO_3_ spiked with 200 ng/mL Iridium (Ir; analytical internal standard, Inorganic Ventures, Christiansburg, VA) for 60 min at 90°C. Deionized water was added to each sample for a final HNO_3_ concentration of 3.5%. Pt content in the sample was analyzed using ICP-MS (Agilent 7500cx, Agilent Technologies, Wilmington, DE, USA) and dedicated software (MassHunter Workstation Software v B.01.01). The results for muscle tissue are reported as Pt concentration per gram of tissue, which was adjusted for variability in the weight of each tissue. As per standard methods, the assumption made that the density of muscle tissue was 1 gram/mL, which allowed for continuity of results to be reported as ng/mL for both plasma and muscle. The lower limit of quantification (LLOQ) for total and unbound Pt in plasma and total Pt in tissues was 1 ng/mL.

### Pharmacokinetic analysis

Non-compartmental pharmacokinetic analyses of total Pt in the plasma was performed using commercially available software (Phoenix^®^ WinNonlin^®^ Software version 6.4, Certara, Princeton, NJ). The pharmacokinetic parameters estimated for total Pt in plasma after SC administration included the area under the curve (AUC) from time 0 to the last time point (AUC_last)_ above the LLOQ, which was calculated using the log-linear trapezoidal method. The elimination rate constant (λz) was estimated from a minimum of three time points during the elimination phase. The remainder of reported parameters were generated using standard non-compartmental equations [21,22] The maximum plasma concentration (C_max_) and time to maximum plasma concentration (T_max_) were determined directly from the data.

### Histological assessment

Tissues samples were collected from the perineal implantation site and from the same anatomical area of the contralateral perineal side, and were fixed in 10% neutral-buffered formalin. Samples of the colon and/or pericolonic skeletal muscle were also collected. Tissues were processed routinely into paraffin for histology and 5 micrometer sections were stained with hematoxylin and eosin. A board-certified veterinary pathologist (KL) reviewed sections for histological lesions and the presence and severity of each of the following categories were individually subjectively scored: inflammation, necrosis, edema, hemorrhage, fibrin and fibrosis. A standard lesion severity scale was used for scoring, where a score of: 0 = no lesion, 1 = minimal, 2 = mild, 3 = moderate, 4 = marked [5].

### Statistical analyses

Data was plotted and not normally distributed, therefore the total Pt in plasma was grouped by day and compared using a nonparametric Kruskal-Wallis ANOVA. The total plasma Pt AUC and total plasma Pt C_max_ results between treatment groups were compared using a nonparametric Kruskal-Wallis ANOVA. A Bonferroni’s correction was used to correct for multiple comparisons. Due to evidence of heteroscedasticity in the residuals of the total plasma Pt C_max_, this data was log-transformed for statistical analysis.

The day 7 total Pt concentrations in plasma and muscle were compared using a nonparametric Wilcoxon test on the paired differences (total Pt in plasma minus total Pt in muscle). The same method was used to compare protein-bound and total plasma platinum. A repeated-measures ANOVA was used to assess differences over time, and between treatment groups.

A Fisher’s Exact test was used to test for homogeneity across the range of histological scores. The data was adjusted for multiple testing using the Bonferroni-Holm correction. Significance was set at *p* < 0.05 (with a corrected cut off of *p* = 0.006) for all analyses. Commercially available statistical software was used for all analyses (SAS version 9.3, SAS Institute Inc, Cary, NC).

## Results

The mean bodyweight of the treatment group rats was 376 ± 48.2 gram (range 310–495 gram). Each rat received 5 mg carboplatin total, for an approximate dose of 13.3 mg/kg BW (range to 10.1–16.1 mg/kg BW).

No wound dehiscence, pain on palpation or exudate was noted on any of the study days for any of the rats. Five out of 6 rats in the CP-μD treatment cohort, 6 out of 6 rats in the CP treatment cohort and 3 out of 3 control rats did not appear to have a significant tissue reaction macroscopically. The colonic and rectal wall macroscopically did not appear thickened, or inflamed in any of the 15 rats (12 treatment and 3 control rats).

### Pharmacokinetic (PK) studies

#### Plasma Pt

**Fig 1:**
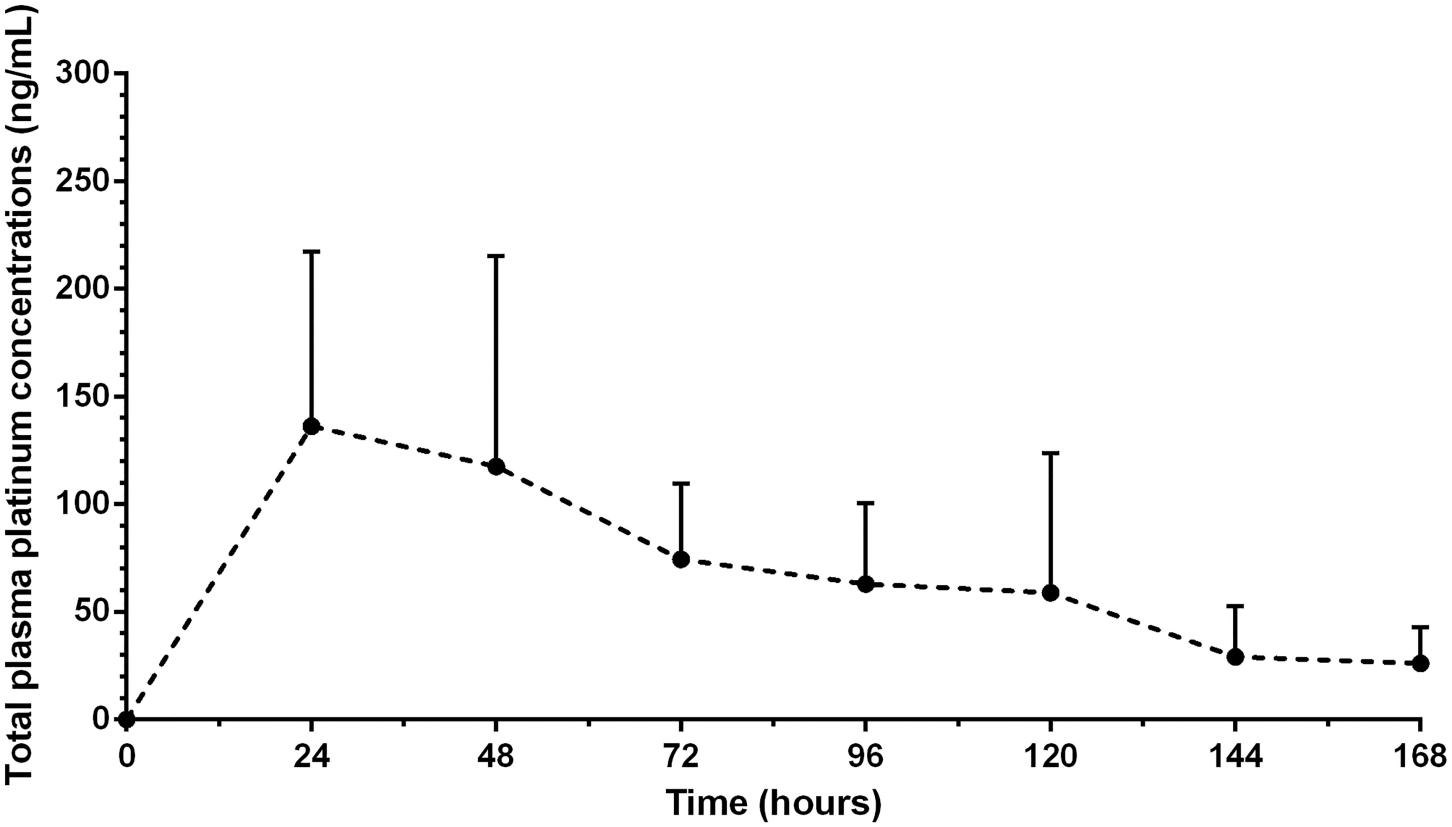
Plasma carboplatin time curve. The concentration versus time curve (mean, standard deviation) of the systemically absorbed carboplatin is given as carboplatinplasma (ng/mL) 7 days. ‘●’ indicates the mean of all treatment groups combined.

No Total Pt was detectable in any of the plasma samples obtained prior to administering carboplatin. Total Pt was detected in all plasma samples from 24 to 168 hours and decreased significantly over time (*p* = 0.01) in both treatment cohorts (Fig 1). There was a significant difference in total Pt exposures in plasma between the two treatment cohorts (*p* = 0.03). The CP cohort had a higher mean total Pt concentration in plasma at 24 hours (201.4 ng/mL) than the CP-μD cohort (82.0 ng/ml).

The pharmacokinetic parameter estimates for total Pt in plasma are presented in Table 1. The pharmacokinetic parameter estimates for the CP and CP-μD cohorts did not significantly differ (*p* = 0.09). The median (range) total Pt C_max_ was 129.4 ng/mL (56.7–349.2 ng/mL) (Table 1). No protein-unbound Pt was detected in any of the plasma samples taken at 168 hours in any rats in either the control or the treatment cohorts.

**Table 1:** Non-compartmental pharmacokinetic parameter of Pt.

		CP cohort (n = 6)	CP-μD cohort (n = 6)	CP and CP-μD cohorts combined (n = 12)
Variable	Units	Median (min–max)	Median (min–max)	Median (min–max)
AUC_last_	ng•h/mL	11,918.1 (7,267.4–21,827.8)	7,938.8 (5,235.5–9,495.3)	9,165.3 (5,235.5–25,997.3)
AUC_%extrapolated_	%	8.4 (4.5–20.9)	9.2 (2.6–19.8)	8.4 (2.6–20.9)
AUMC_last_	ng•h^2^/mL	732,675.7 (423,764.3–1,803,490.4)	599,912.58 (355,037.2–701,699.9)	655,658.2 (355,037.2–1,803,490.4)
Pt C_max_	ng/mL	178.2 (112.3–349.2)	99.0 (56.7–148.3)	129.4 (56.7–349.2)
Half-lifeλz	h	33.4 (22.9–75.3)	34.2 (16.5–68.9	34.0 (16.5–75.3)
λz	1/h	0.02 (0.0092–0.0302)	0.0203 (0.0100–0.419)	0.0203 (0.0092–0.0419)
MRT_last_	h	61.5 (57.9–69.4)	73.5 (65.4–85.8)	67.8 (57.9–85.8)
T_max_	h	24.0 (24.0–48.0)	36 (24–120)	24 (24.0–120.0)

Non-compartmental pharmacokinetic parameter estimates of carboplatin (Pt) in plasma after perineal SC administration in poloxamer copolymer gel. This table includes the results (median, minimum–maximum) for the individual cohorts, as well as the grouped results (median, minimum–maximum) for the CP [n = 6] and CP-μD [n = 6] cohorts [n = 12]. These groups were combined to allow a more robust analysis of the results. AUC, area under the curve from t = 0 to the last time point, AUC_%extrapolated_ % AUC extrapolated to infinity, AUMC_last_ = area under the first moment curve to the last time point, C_max_ = maximum plasma concentration, half-life_λz_ = terminal half-life, λz = elimination rate constant, MRT_last_ = mean residence time to the last time point, T_max_ = time to maximum plasma concentration.

#### Wound bed

**Fig 2:**
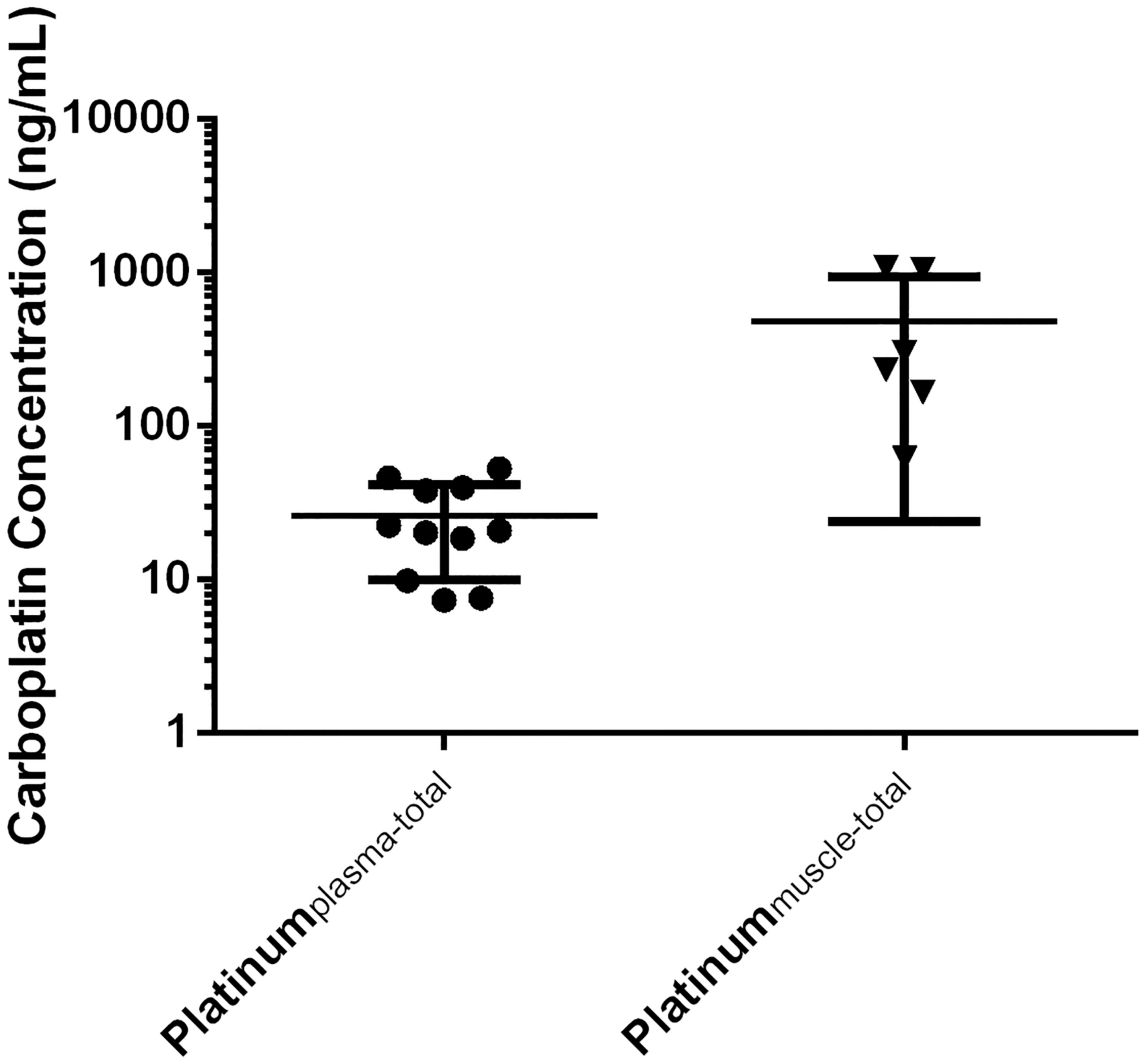
Concentration of total Pt in plasma and total Pt in muscle at 168 hrs. The carboplatin concentration (ng/mL) is plotted on the Y-axis in a logarithmic scale. ‘●’ and ‘▼’ represent the Ptplasma (n = 11)and Ptmuscle (n = 6) of every individual rat, respectively.

All muscle samples from the CP treatment cohort (n = 6) contained total Pt [median 268 ng/ml (range 61.7–1083.5 ng/mL)], 6.7 times higher compared to the total Pt in plasma (40ng/ml) in the treatment group (Fig 2). This difference did not reach statistical significance (*p* = 0.059).

### Histology

**Fig 3:**
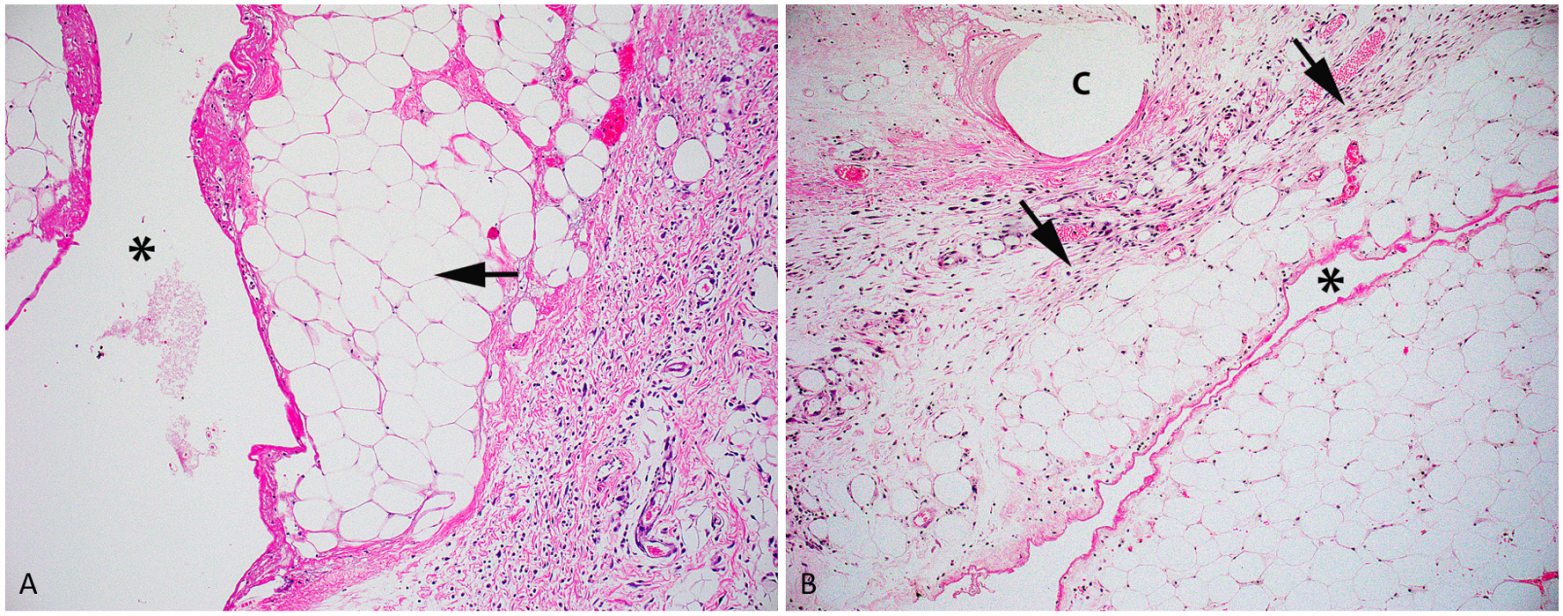
Histology of the subcutaneous perineal implantation site. A Shows a photomicrograph of the subcutaneous perineal surgical implantation site of rat from the CP cohort. Minimal necrosis of adipose (score = 1) (arrow) is present adjacent to the implantation site (asterisk). B Shows a photomicrograph of the subcutaneous perineal surgical implantation site of a rat from the CP-μD cohort. Minimal fibrosis (score = 1) (arrows) is present adjacent to the implantation site (asterisk) after receiving carboplatin and poloxamer 407 gel and a μD catheter (C) at the site.

All colonic specimens from all rats were scored ‘0’ for all categories. Inflammation was seen in surgery side wound bed tissue specimens (Fig 3) The median histologic scores for the operated side of the control group were different from zero for inflammation: 2 (range 2–2), necrosis: 1 (range 1–1), and fibrosis 2 (range 2–2) (Table 2). The scores of the operated and the non-operated side were converted to an intra-rat difference [score of the operated side—the score of the non-operated side]. These intra-rat differences are shown in Table 2 for all parameters assessed. The intra-rat difference of the scores did not significantly differ between any of the groups (treatment or control groups) (Table 2).

**Table 2:** Histological scores.

	Control (n = 3)		CP cohort (n = 6)			CP-μD cohort (n = 6)		
	Score R	Difference R vs L	Score R	Difference R vs L	*p*	Score	Difference R vs L	*p*
Inflammation	2 (2–2)	2 (2–2)	1.5 (1–2)	1.5 (1–2)	1.0	2 (1–2)	2 (1–2)	1.0
Necrosis	1 (1–1)	1 (1–1)	1 (1–3)	1 (1–3)	1.0	1 (0–2)	1 (0–2)	1.0
Hemorrhage	0 (0–0)	0 (0–0)	0 (0–3)	0 (0–3)	1.0	0 (0–0)	0 (0–0)	*
Edema	0 (0–0)	0 (0–0)	1 (1–3)	1 (1–3)	0.07	1.5 (1–3)	1.5 (1–3)	0.18
Fibrin	0 (0–0)	0 (0–0)	1.5 (1–3)	1.5 (1–3)	0.18	1 (0–3)	1 (0–3)	0.64
Fibrosis	2 (2–2)	2 (2–2)	1 (1–2)	1 (1–2)	0.19	1 (1–2)	1 (1–2)	0.66

The median and ranges for the histologic scoring of the operated side (right side) are shown for the control group and all three groups separately, as well as all treated rats combined. The difference between the scores of the operated side vs the contralateral side (same rat) are shown “difference R vs L” and were used to compare between treatment cohorts and control rats (Fisher’s exact test) for which the p values are shown. CP: Carboplatin in poloxamer gel, μD: microdialyis catheter,

*All zero.

## Discussion

The first hypothesis of the current study was that carboplatin in poloxamer copolymer gel instilled into the perineal subcutaneous tissues will provide a sustained local presence of carboplatin leading to high local tissue concentrations but low plasma concentrations. We identified prolonged local tissue concentrations of carboplatin, at a higher level than concurrent 168 hour plasma levels. The second hypothesis of the current study was that subcutaneously implanted carboplatin-poloxamer at a concentration of 5mg/ml does not cause local tissue necrosis in the perineal area. We did not identify any gross or histologic evidence of tissue necrosis or wound complications after subcutaneous implantation of 5mg carboplatin in poloxamer 407 in healthy rats, supporting the safety profile of a locally instilled carboplatin in poloxamer copolymer gel into a subcutaneous perineal wound bed.

Overall these results suggest that carboplatin in poloxamer copolymer gel can act as a depot with sustained presence and local release of carboplatin for a minimum of 7 days without major incisional complications or tissue necrosis.

We chose to examine the highest possible local carboplatin concentration that we could instill in the wound as we aimed to examine the toxicity and tissue safety of locally-instilled carboplatin. The amount of 5mg carboplatin in a total volume of 1ml was chosen as the highest concentration of commercially available carboplatin (10mg/ml) that can be added to poloxamer copolymer gel and still allow gelatination of the compound, which would best allow for the assessment of tissue damage in the *in vivo* portion of the study. A higher ratio of carboplatin to poloxamer copolymer gel was assessed but did not fully convert to a gel state (unpublished data), which would have potentially increased the risk for loss of the compound through the incision line. Due to space limitations in the subcutaneous space, it was not possible to instill more than one ml of carboplatin-poloxamer per rat. Rats in this study therefore received 5mg carboplatin total dose per rat, or approximately 13.3 μg/g BW [= 13 mg/kg BW, or 2.17mg/m2] [23] carboplatin on Day 0. In comparison, clinically used IV doses of carboplatin are 300 mg/m2 (dogs) and 240–260 mg/m2 (cats) [24] of which our administered dose falls well below. Total plasma Pt levels rapidly declined after approximately 24 hours (11 rats) or 48 hours (one rat), and were 6.7-fold less than the corresponding muscle tissue Pt levels at 168 hours. No free (unbound) carboplatin was found at 168 hours in the plasma of any of the rats. A prior rodent study using a carboplatin bolus IV of 20 and 30mg/kg, and showed peak plasma concentrations of 13.2 and 31.9 μg/ml within 60 minutes [25] with a t_1/2_ of 139.7 and 120.5minutes, respectively. Therefore, there is a possibility that the maximum plasma Pt concentration was reached earlier than 24 hours post-implantation and therefore not captured at the 24hr time point. Daily (q24 hour) sampling points were chosen as, to date, no *in vivo* absorption data of subcutaneous carboplatin in poloxamer copolymer gel exist. The goal of this study was not to assess the maximum level of systemic absorption, but rather to obtain data on the concentration and duration of local tissue Pt in the wound bed, and to investigate the effects of SC implantation of carboplatin in poloxamer copolymer gel on the local tissues. However, given the results of this study, the addition of more sampling time points within the first 24 hours could allow us to further define the maximum plasma Pt concentration.

The persistent low levels of total plasma Pt concentrations most likely can be attributed to the residual protein bound fraction of carboplatin rather than the continued absorption of the locally implanted carboplatin. Carboplatin has a high % of irreversible protein binding after 24 hours, but it is lower and occurs later than cisplatin protein binding: 90–95% for cisplatin versus 40–50% for carboplatin [26,27] while a different reference cites 85–89% protein binding after 24 hours (www.Medsafe.govt.nz). In addition, the elimination half-life of IV administered carboplatin in rats has been reported to be relatively short at 128±75.7 min [25] which is in contrast to the prolonged half-life we estimated following SC administration in the poloxamer copolymer gel. Ideally, unbound and bound platinum levels would have been measured at all time points for all rats, but that would have increased the volume of blood needed for sampling which the authors deemed to be unethical given the size of the rats used in this study (0.3ml of blood was taken for each sample in the present study) at all time points.

The median of the measured local tissue total Pt concentrations at 168 hours was 233.7 ng/mL. While the effective *in vivo* tissue concentration of carboplatin is not known, one *in vitro* study has found the 72 hour *in vitro* IC_50_ of carboplatin for malignant mammary tumors to be 30.5 μM (11.3 μg/mL) [28], while the IC_50_ for carboplatin in canine osteosarcoma cell lines was 100 μM [29] Both of these concentrations are well above the concentrations reached in the present study. No data currently exists for *in vivo* tumor IC target dose for ASAGAC, nor is any information known regarding the levels of carboplatin necessary to obtain 50% cytotoxicity after 168 hours of exposures *in vitro* or *in vivo*. Little is known about tissue concentrations achieved in surgical sites after systemic administration of chemotherapeutics. One previous study has shown that tumoral tissue concentrations after IV administration of 3 mg/kg of cisplatin in mice was 0.84 μg/g after 1hour [20] While the 168 hour tissue Pt levels found in the current study were below this concentration, we did find prolonged (168 hour) exposure to carboplatin at the site of the wound.

Based on daily wound assessments and clinical appearance, perineal subcutaneous carboplatin instillation in rats using a 5mg/ml concentration, was not associated with tissue necrosis or adverse effects. While it is possible that the histological assessment at 168hrs missed earlier signs of necrosis and/or inflammation, no wound related issues or complications were noted on our daily examination, providing an argument that any potentially missed histological findings were self-limiting and not of clinical relevance.

Subjectively, only a small amount of tension was needed to separate the incision after formalin fixation and skin suture removal, although no dehiscence had been noted *in vivo*. Unfortunately, we were unable to specifically score for the incision line integrity on histology, as the manipulation from removing the stainless steel sutures and trimming the samples distorted the incisions. Future studies to investigate this subjective finding further could include tensile strength testing on a subset of the specimens, not destined for histological assessment. In addition, while no necrosis was found histologically, the treatment rats did have a higher score for edema in the surgery site. This might be caused by a foreign body reaction (due to the presence of the microdialysis probe), or could be a reaction to the carboplatin that was not severe enough to induce a tissue necrosis or wound healing complications.

The results of the total muscle Pt concentrations suggest that carboplatin in poloxamer copolymer gel could be used *in vivo* providing direct tissue exposure to carboplatin without significant local effects or systemic absorption. Future *in vivo* directions should include proof of tissue safety in target species prior to clinical use, in addition to a better understanding of the dose and time of exposure needed by obtaining targeted IC_50_ data and a more extensive assessment of local tissue Pt concentrations.

Subcutaneous carboplatin in a poloxamer copolymer gel (5 mg/ml) in a perineal location was not associated with clinical adverse effects in an *in vivo* rodent model, but caution and use of a slow or non-absorbable suture is advised in clinical patients.

## Supporting information

S1 File PK data. (XLSX)

S2 File Raw data for individual Pt assays and histology scoring. (XLSX)

S3 File Histology scoring data. (XLSX)

S4 File ARRIVE guidelines checklist. (PDF)
